# The underestimated burden of postoperative pulmonary complications in emergency laparotomy: a propensity score-matched analysis

**DOI:** 10.3389/fmed.2025.1733029

**Published:** 2025-12-15

**Authors:** Anca-Laura Amati, Nicoleta Negruta, Romina Ebert, Niklas Kümmel, Moritz Fritzenwanker, Matthias Wolff, Sebastian Petzoldt, Martin Reichert, Andreas Hecker

**Affiliations:** 1 Department of General, Visceral, Thoracic and Transplant Surgery, University Hospital of Giessen, Justus-Liebig University Giessen, Giessen, Germany; 2 Institute of Medical Microbiology, Justus-Liebig-University Giessen, Giessen, Germany; 3 Department of Anesthesiology, Intensive Care Medicine and Pain Therapy, University Hospital of Giessen, Justus-Liebig University Giessen, Giessen, Germany

**Keywords:** emergency laparotomy, postoperative pulmonary complications, risk prediction, multidrug-resistant organisms, survival analysis

## Abstract

**Introduction:**

Emergency laparotomy (EL) remains one of the highest-risk procedures in general surgery, characterized by substantial postoperative morbidity and mortality. Despite major advances in perioperative medicine, current enhanced-recovery and infection-prevention protocols are largely derived from elective surgical cohorts and are seldom validated in emergency settings. This lack of EL-specific data represents a critical gap in establishing evidence-based perioperative guidelines for this uniquely vulnerable patient population. Among postoperative complications, pulmonary complications (PPCs) are the most frequent and consequential, affecting 20–40% of patients and significantly impairing recovery and survival. This study aimed to identify preoperative predictors of PPCs and their impact on postoperative mortality, highlighting potentially actionable targets within the constraints of emergency surgical care.

**Methods:**

A total of 928 EL patients were analyzed. To control for non-modifiable demographics and comorbidities, propensity score matching generated two cohorts of 328 patients each—those with and without PPCs. Univariate and multivariate logistic regression identified independent preoperative predictors of PPCs, and survival analyses assessed their association with postoperative mortality.

**Results:**

Mortality was significantly higher in patients with PPCs (42.1%) than in those without (11.9%). Independent preoperative predictors of PPCs included ASA score (*p* = 0.0004), hemoglobin level (*p* = 0.0340), C-reactive protein-to-albumin ratio (CAR) (*p* = 0.0001), and colonization with multidrug-resistant organisms (MDROs) on preoperative screening (*p* = 0.0420). Each of these factors also predicted reduced postoperative survival. Notably, 67.3% of MDROs were not covered by initial empiric antibiotic regimens, and 28.6% of Gram-negative MDROs detected preoperatively were later isolated from the airways of patients who developed PPCs. PPC patients colonized with MDROs had a 47% higher 90-day mortality risk compared with non-colonized counterparts (HR 1.46, 95% CI 0.99–2.15).

**Conclusion:**

PPCs are the most frequent and deadly complications following EL, and their occurrence substantially worsens survival. Among identified predictors, Gram-negative MDRO colonization represents the most clinically actionable target. Tailoring empiric antibiotic therapy for high-risk, colonized EL patients could represent a pivotal step toward evidence-based, condition-specific perioperative guidelines aimed at reducing pulmonary complication–related mortality.

## 1 Introduction

Emergency laparotomy (EL) is one of the most frequently performed yet highest-risk surgical procedures worldwide. Compared to elective abdominal surgery, EL is associated with a significantly higher complication rate—exceeding 50%—and a mortality rate up to five times greater (1, 2). Efforts by the Enhanced Recovery After Surgery (ERAS) Society (3, 4) have marked important progress toward evidence-based emergency perioperative care. Yet, the scarcity of high-quality, EL-specific evidence continues to impede the development of robust guidelines.

Unlike elective patients, who typically undergo surgery in an optimized physiological state, EL patients face the compounded physiological stress resulting from both the acute underlying pathology and the added burden of surgical trauma (5). This dual insult disrupts neuroendocrine, metabolic, and immune homeostasis, heightening susceptibility to complications that cannot be reliably predicted using elective surgery data (6). Numerous studies have identified the emergent nature of surgery itself as an independent risk factor for adverse outcomes, including postoperative pulmonary complications (PPCs), which are among the most common complications following EL (1, 7, 8).

In elective abdominal surgery, PPC incidence ranges from <1 to 9%, depending on definitions and cohort characteristics (9–11). In stark contrast, the multicenter prospective ALPINE study reported a 48% PPC incidence in EL patients (12). These complications—most commonly respiratory failure, atelectasis, and infection—substantially impair recovery and are associated with increased intensive care unit (ICU) admissions, reintubation rates, prolonged mechanical ventilation, and higher mortality (8, 9).

Opportunities for PPC prevention in EL are limited, as preoperative strategies commonly used in elective settings—such as respiratory physiotherapy or structured prehabilitation programs—are largely unfeasible in the acute context (13, 14). Consequently, intraoperative and early postoperative management—including ventilation strategies, pulmonary care bundles, and timely antibiotic therapy when a respiratory infection is suspected —play a central role. Yet, evidence from elective contexts cannot be applied uncritically: for instance, lung-protective ventilation failed to reduce PPC incidence in EL patients in the ALPINE study (12, 15).

Antibiotic management of PPCs in EL remains understudied (16). In a cohort of 7,275 abdominal surgery patients, nearly half of those who developed postoperative pneumonia received inappropriate antibiotic treatment—significantly increasing pneumonia-specific mortality (17). The challenge is exacerbated by ongoing uncertainty around optimal respiratory sampling methods in critically ill patients and the rising prevalence of antimicrobial resistance (18). Empiric antibiotic selection requires both accurate patient-specific risk stratification and familiarity with local resistance patterns (19).

Notably, preoperative colonization with multidrug-resistant organisms (MDROs) has been linked to higher complication rates, including PPCs, across various surgical cohorts (20–22). However, its specific role in EL remains unclear.

Addressing these knowledge gaps is essential. A nuanced understanding of how acute pathophysiological and surgical stressors contribute to PPC risk may enable the development of more targeted, EL-specific management strategies. In particular, identifying modifiable risk factors and elucidating the impact of MDRO colonization could inform preventive measures, optimize the timing of interventions, and support improved antibiotic stewardship in EL patients.

Accordingly, this study had two primary objectives: to identify potentially modifiable pre- and intraoperative predictors of PPCs in the setting of EL, and to characterize the spectrum of MDRO colonization most likely to impair postoperative pulmonary recovery.

## 2 Methods

This single-center, observational study retrospectively analyzed data from 928 patients who underwent EL between January 2014 and December 2020 at the Department of General, Visceral, Thoracic, and Transplant Surgery, University Hospital of Giessen. All data were drawn from prospectively maintained institutional databases.

The study was conducted in accordance with the Declaration of Helsinki and approved by the local ethics committee of the Medical Faculty at the University of Giessen (Approval No. 20/22). Given the retrospective design, the requirement for written informed consent was waived. Data collection, manuscript preparation, and submission adhered to the Committee on Publication Ethics (COPE) and the Strengthening the Reporting of Observational Studies in Epidemiology (STROBE) guidelines (23).

### 2.1 Patient selection

Inclusion criteria comprised all adult patients (≥18 years) who underwent EL within the specified timeframe. Excluded were laparoscopic emergency procedures, diagnostic ELs limited to assessing bowel viability or those that ruled out an intra-abdominal pathology as the cause of the patient’s critical condition (negative laparotomy), as well as all trauma-related laparotomies.

### 2.2 Data collection

Collected data included baseline demographics, comorbidities, and the American Society of Anesthesiologists (ASA) classification (categories 1–5), as well as perioperative laboratory parameters reflecting systemic inflammation [leukocyte count, C-reactive protein (CRP)], liver function (albumin, bilirubin), and renal function (creatinine).

Microbiological data included results from preoperative MDRO screening and postoperative airway sampling, together with the corresponding antibiotic resistance profiles. Nasal, oral, and anal swabs for MDRO detection were obtained either preoperatively or, at the latest, immediately after surgery upon ICU admission. Postoperative airway sampling was not performed routinely but selectively in patients with clinical and radiologic suspicion of PPCs, using either invasive or non-invasive methods such as bronchial sampling, bronchoalveolar lavage, or endotracheal aspiration (19).

MDROs were classified according to the criteria established by the German Commission for Hospital Hygiene and Infection Prevention (KRINKO) at the Robert Koch Institute (RKI) (24). This classification included methicillin-resistant *Staphylococcus aureus* (MRSA), vancomycin-resistant enterococci (VRE), and multidrug-resistant Gram-negative bacteria (MRGN), such as—but not limited to—extended-spectrum β-lactamase (ESBL)-producing *Enterobacterales*. MRGNs were further categorized as 3MRGN or 4MRGN, defined by resistance to three or four key antibiotic classes—acylureidopenicillins, third- and fourth-generation cephalosporins, fluoroquinolones, and carbapenems—according to classification standards from both KRINKO and the World Health Organization (WHO) (24, 25).

Operative details were extracted from narrative surgical reports and standardized using the synoptic EL reporting framework by Elamin et al. (26). Recorded parameters included the location of the primary pathology (upper gastrointestinal tract, small bowel, large bowel, other), bowel resection (none, upper gastrointestinal tract, small bowel, small and large bowel, large bowel), anastomosis type (none, upper gastrointestinal tract, small bowel, small-to-large bowel, large bowel), additional surgical procedures (yes/no), operative duration (minutes), and intraoperative blood loss (mL).

### 2.3 Outcome measures

The primary outcome was the incidence of PPCs within 7 days after EL, defined according to the European Perioperative Clinical Outcome (EPCO) criteria (27). These include respiratory failure, infection, atelectasis, aspiration, bronchospasm, and pneumothorax. PPCs were assessed through chart reviews, ventilation records, imaging (X-ray/CT), and bronchoscopy findings.

Secondary outcomes included the incidence of major surgical complications (Clavien–Dindo grade ≥IIIb) (28), the length of hospital and ICU stay, and 90-day all-cause mortality.

### 2.4 Statistical analysis

Statistical analyses were performed using GraphPad Prism v10 (GraphPad Software, San Diego, CA, United States) and R v4.3.2 (The R Foundation for Statistical Computing). Patients were stratified based on the occurrence of PPCs within the first week following EL into two groups: the PPC group and the no-PPC group.

To reduce confounding, propensity score matching (PSM) was applied using a 1:1 nearest-neighbor algorithm without replacement in R. Matching variables included age, sex, body mass index (BMI), and comorbidities such as hypertension, cardiovascular, pulmonary, hepatic, renal, or inflammatory diseases, diabetes, immunosuppression, and malignancy.

Group comparisons for continuous variables were conducted using the Mann–Whitney *U* test, while categorical variables were analyzed using Fisher’s exact or chi-square tests. Data are presented as medians with interquartile ranges (IQRs) or as absolute numbers and percentages [*n* (%)]. A two-tailed *p*-value <0.05 was considered statistically significant.

To identify factors associated with PPCs, univariate logistic regression analyses were performed. The predefined variables included the ASA classification (categories 1–5), preoperative hemoglobin concentration (g/L), the CRP/albumin ratio (CAR), preoperative creatinine level (mg/dL), and preoperative MDRO colonization (yes/no). Additionally, the localization of the leading pathology and the type of surgical procedure (as defined above) were included as categorical variables. Intraoperative blood loss (mL) and duration of surgery (minutes) were entered as continuous variables. Variable selection was based on differences observed between the two propensity score–matched groups, as well as on predictors previously described for elective surgery (9). Variables demonstrating significant associations in univariate analyses were subsequently entered into a multivariate logistic regression model. Multicollinearity was assessed via variance inflation factors (VIF), applying a conservative threshold of 2.5.

Kaplan–Meier estimates were used to evaluate 90-day survival probabilities. Group differences were analyzed using the log-rank test, from which hazard ratios (HRs) and 95% confidence intervals (CIs) were derived.

The optimal cutoff value for the CAR in predicting 90-day mortality was determined using the Youden index (29), calculated with the “cutpointr” package in R.

## 3 Results

### 3.1 Baseline characteristics of the total and the propensity score-matched patient cohort

During the study period, 928 patients underwent EL, of whom 328 (35.3%) developed PPCs within the first seven postoperative days. Patients who developed PPCs were significantly older (median age: 70 vs. 64 years, *p* < 0.001) and had a higher prevalence of comorbid conditions, including hypertension, diabetes mellitus, and chronic cardiac, pulmonary, hepatic, and renal diseases. There were no significant differences between groups in the rates of systemic immunosuppression, chronic inflammatory diseases, or previous malignancy (Table 1).

**Table 1:** Baseline characteristics of the no-PPC and PPC patient cohorts before and after PSM.

Variable	Before PSM			After PSM		
	No-PPC (*n* = 600)	PPC (*n* = 328)	*p* value	No-PPC (*n* = 328)	PPC (*n* = 328)	*p*-value
Age (years; IQR)	64 (51, 76)	70 (59, 79)	**<0.001**	70 (57, 78)	70 (59, 79)	0.8
Sex (*n* males; %)	329 (55%)	192 (59%)	0.3	196 (60%)	192 (59%)	0.8
BMI (kg/m^2^; IQR)	25 (22, 29)	26 (22, 29)	0.2	26 (23, 30)	26 (22, 29)	0.7
Comorbidities						
Arterial hypertension (*n* patients; %)	89 (15%)	69 (21%)	**0.016**	68 (21%)	69 (21%)	>0.9
Chronic cardiac disease (*n* patients; %)	184 (31%)	167 (51%)	**<0.001**	160 (49%)	167 (51%)	0.6
Chronic pulmonary disease (*n* patients; %)	100 (17%)	89 (27%)	**<0.001**	72 (22%)	89 (27%)	0.12
Diabetes mellitus (*n* patients; %)	88 (15%)	92 (28%)	**<0.001**	79 (24%)	92 (28%)	0.2
Chronic kidney disease (*n* patients; %)	92 (15%)	120 (37%)	**<0.001**	91 (28%)	120 (37%)	0.015
Chronic liver disease (*n* patients; %)	50 (8.3%)	44 (13%)	**0.014**	46 (14%)	44 (13%)	0.8
Systemic immunosuppression (*n* patients; %)	15 (2.5%)	12 (3.7%)	0.3	12 (3.7%)	12 (3.7%)	>0.9
Chronic inflammatory disease (*n* patients; %)	59 (9.8%)	39 (12%)	0.3	41 (13%)	39 (12%)	0.8
Previous malignoma (*n* patients; %)	242 (40%)	129 (39%)	0.8	130 (40%)	129 (39%)	>0.9

Patients who underwent emergency laparotomy (EL) without postoperative pulmonary complications (PPCs) during the first postoperative week were compared with those who developed PPCs, before and after propensity score matching (PSM) for demographic traits and chronic illnesses. Data are presented as median with interquartile range (IQR) or as number of patients (*n*) with percentages (%). BMI, body mass index. *p* < 0.05 are indicated in bold.

To isolate the impact of acute pathology and surgical trauma while minimizing the influence of non-modifiable preconditions, PSM was performed, resulting in 328 matched pairs with balanced demographics and comorbidity profiles (Table 1).

### 3.2 Perioperative characteristics of the propensity matched patient cohorts

In the PSM cohort, we analyzed preoperative and intraoperative parameters influenced by the acute surgical pathology and the surgical procedure itself, aiming to identify actionable risk factors for the development of PPCs.

Preoperative physical status, reflected by the ASA score, was significantly worse in patients who subsequently developed PPCs (*p* < 0.0001). While preoperative white blood cell counts were similar between groups, preoperative CRP levels were markedly elevated in the PPC group (median: 148.5 vs. 63.9 mg/L, *p* < 0.0001), as was the CAR (median: 4.6 vs. 1.8, *p* < 0.0001), a composite inflammatory marker validated in critically ill patients (30–32).

Patients developing PPCs also had significantly lower preoperative hemoglobin levels and higher creatinine concentrations. Furthermore, colonization with MDROs was more common in the PPC group (25.3% vs. 13.1%, *p* = 0.0001).

The primary surgical pathology most commonly involved the small bowel in both groups; however, upper gastrointestinal and large bowel conditions were disproportionately represented among PPC patients. Accordingly, resections involving these regions were more frequently performed in the PPC group. Despite these anatomical differences, the distribution of gastrointestinal anastomoses and sutures within the GI tract remained balanced between the two groups. Surgical duration was also significantly longer in the PPC cohort.

Postoperatively, PPC patients required markedly prolonged ventilatory support following initial intubation (median time to extubation: 14 vs. 3 h, *p* < 0.0001), which contributed to extended ICU stays. These patients experienced a higher incidence of severe complications (Clavien–Dindo grade ≥IIIb), including the need for surgical reinterventions. Notably, the 90-day mortality rate in the PPC group was 42.1%, in contrast to 11.9% in the no-PPC group (Table 2).

**Table 2:** Perioperative characteristics of the propensity-matched no-PPC and PPC patient groups.

Variable	No-PPC (*n* = 328)	PPC (*n* = 328)	*p*-value
Preoperative parameters			
ASA (score, IQR)	3 (3, 3)	3 (3, 4)	**<0.0001**
Leukocyte count (×10^9^/L, IQR)	10.4 (7.2, 15.2)	10.5 (6.6, 17.7)	0.6046
Hemoglobin (g/L, IQR)	117 (100, 136)	105 (92, 125)	**<0.0001**
Thrombocyte count (×10^9^/L, IQR)	250 (187, 330)	246 (175, 324)	0.4378
CRP (mg/L, IQR)	63.9 (18.3, 165.3)	148.5 (49.2, 148.5)	**<0.0001**
Albumin (g/L, IQR)	34.3 (29.1, 39.7)	30.20 (26.9, 35.1)	**<0.0001**
CRP/Albumin (ratio, IQR)	1.8 (0.5, 5.2)	4.6 (1.5, 8.1)	**<0.0001**
Creatinine (mg/dL, IQR)	1.0 (0.7, 1.6)	1.2 (0.8, 2.0)	**0.0039**
Bilirubin (mg/dL, IQR)	0.7 (0.4, 1.1)	0.7 (0.4, 1.1)	0.5665
MDRO colonization (*n* patients, %)	43 (13.1%)	83 (25.3%)	**0.0001**
Surgery characteristics			
Localization of leading pathology			**0.0014**
Upper GI tract (*n* patients, %)	46 (14%)	62 (18.9%)	
Small bowel (*n* patients, %)	184 (56.1%)	136 (41.5%)	
Large bowel (*n* patients, %)	97 (29.6%)	127 (38.7%)	
Other (*n* patients, %)	1 (0.3%)	3 (0.9%)	
Surgical procedure: bowel resection			**0.0044**
None (*n* patients, %)	167 (50.5%)	132 (40.1%)	
Upper GI (*n* patients, %)	7 (2.1%)	17 (5.2%)	
Small bowel (*n* patients, %)	62 (18.7%)	50 (15.2%)	
Small and large bowel (*n* patients, %)	38 (11.5%)	49 (14.9%)	
Large bowel (*n* patients, %)	57 (17.2%)	81 (24.6%)	
Bowel anastomosis			0.2951
None (*n* patients, %)	149 (44.1%)	158 (45.7%)	
Upper GI (*n* patients, %)	42 (12.4%)	59 (17.1%)	
Small bowel (*n* patients, %)	84 (24.9%)	76 (22%)	
Small to large bowel (*n* patients, %)	37 (10.9%)	35 (10.1%)	
Large bowel (*n* patients, %)	26 (7.7%)	18 (5.2%)	
Additional surgical procedure (*n* patients, %)	66 (20.1%)	79 (24.1%)	0.2588
Intraoperative blood loos (mL, IQR)	150 (100, 300)	200 (100, 500)	**<0.0001**
Duration of surgery (min, IQR)	103 (73, 147)	120.5 (84.7, 160)	**0.0026**
Postoperative outcomes			
Ventilation time until initial extubation (hours, IQR)	3 (0, 7.5)	14 (4.7, 72.7)	**<0.0001**
Tracheotomy (*n* patients, %)	4 (1.2%)	56 (17%)	**<0.0001**
Length of ICU stay (days, IQR)	2 (1, 4)	8 (3, 20)	**<0.0001**
Surgical complications ≥CD IIIb^*^ (*n* patients, %)	71 (21.7%)	126 (38.4%)	**<0.0001**
Surgical reintervention (*n* patients, %)	44 (13.4%)	91 (27.7%)	**<0.0001**
ICU re-admission (*n* patients, %)	56 (17.1%)	60 (18.3%)	0.7589
Length of hospital stay (days, IQR)	12 (8, 20)	18 (9, 34.7)	**<0.0001**
90-day mortality (*n* patients, %)	39 (11.9%)	135 (41.2%)	**<0.0001**

Pre- and intraoperative data of patients without or with postoperative pulmonary complications (PPC), matched for demographic and chronic disease characteristics, were compared. Data are presented as median with interquartile range (IQR) or as number of patients (*n*) with percentages (%). ^*^According to the Clavien–Dindo (CD) classification of surgical complications. ASA, American Society of Anesthesiologists; CRP, C-reactive protein; MDRO, multidrug-resistant organism; GI, gastrointestinal; ICU, intensive care unit. *p* < 0.05 are indicated in bold.

### 3.3 Risk factors for PPC development

Univariate logistic regression identified several preoperative and intraoperative predictors of PPC development following EL. On multivariable analysis, independent predictors included elevated CAR (*p* = 0.0001), higher ASA score (*p* = 0.0004), lower preoperative hemoglobin (*p* = 0.0340), and MDRO colonization (*p* = 0.0420). In contrast, intraoperative variables such as surgical duration, blood loss, and type of procedure were not significantly associated with an elevated PPC risk (Table 3).

**Table 3:** Univariate and multivariate analysis of preoperative and intraoperative factors for predicting PPC development.

Variable	Univariate		Multivariate	
	OR (95% CI)	*p*-value	OR (95% CI)	*p*-value
ASA score	2.091 (1.641–2.688)	**<0.0001**	1.657 (1.255–2.206)	**0.0004**
Preoperative hemoglobin levels	0.9818 (0.9750–0.9885)	**<0.0001**	0.9906 (0.9818–0.9992)	**0.0340**
Preoperative CRP/albumin ratio	1.134 (1.088–1.184)	**<0.0001**	1.095 (1.045–1.148)	**0.0001**
Preoperative creatinine level	1.072 (0.9967–1.208)	0.2316		
Preoperative MDRO colonization	2.245 (1.504–3.393)	**<0.0001**	1.657 (1.023–2.717)	**0.0420**
Localization of leading pathology	1.158 (0.9978–1.417)	0.1092		
Surgical procedure: bowel resection	0.9206 (0.8185–1,035)	0.1663		
Intraoperative blood loss	1.000 (1.000–1.001)	**0.0121**	1.000 (0.9999–1.001)	**0.2022**
Duration of surgery	1.002 (0.9996–1.004)	0.1053		

Preoperative and intraoperative factors significantly associated with postoperative pulmonary complication (PPC) development in the univariate analysis (*p* < 0.05) were included in the multivariate logistic regression model. OR, odds ratio; CI, confidence interval; ASA, American Society of Anesthesiologists; CRP, C-reactive protein; MDRO, multidrug-resistant organism. *p* < 0.05 are indicated in bold.

### 3.4 Impact of preoperative factors on 90-day survival

**Figure 1:**
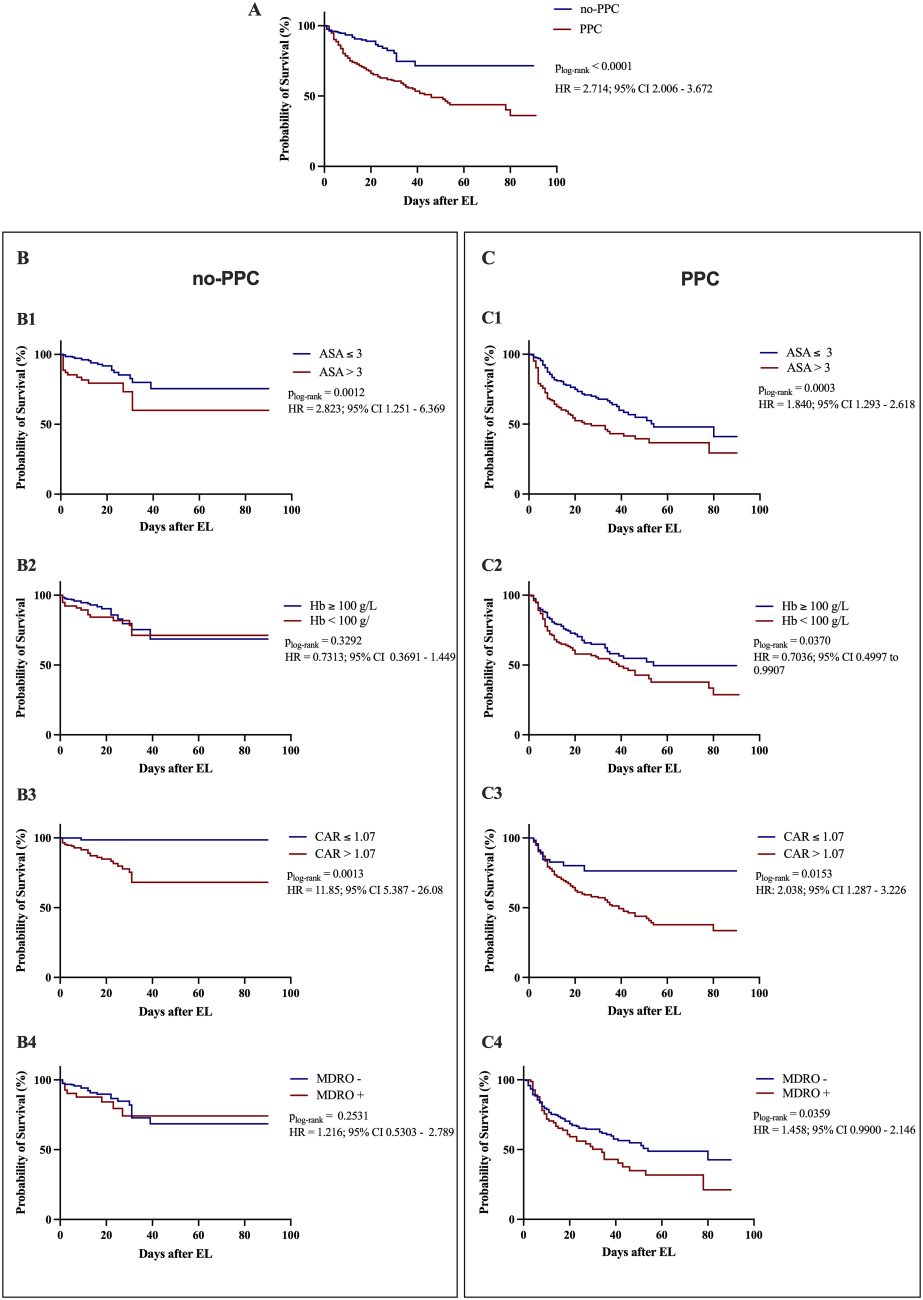
Kaplan–Meier survival curves for patients with or without PPCs, stratified by identified risk factors. **(A)** Ninety-day survival of patients with and without postoperative pulmonary complications (PPCs) following emergency laparotomy. **(B)** Ninety-day survival of patients without PPCs, stratified by **(B1)** American Society of Anesthesiologists (ASA) physical status, **(B2)** preoperative hemoglobin (Hb) level, **(B3)** preoperative C-reactive protein-to-albumin ratio (CAR), and **(B4)** colonization status with multidrug-resistant organisms (MDROs). **(C)** Ninety-day survival of patients with PPCs, stratified by **(C1)** ASA score, **(C2)** preoperative Hb level, **(C3)** preoperative CAR, and **(C4)** MDRO colonization. The log-rank test was used to assess survival differences between groups. Hazard ratios (HRs) with corresponding 95% confidence intervals (CIs) are shown for each comparison, contrasting the group represented in red with the group in blue.

PPC development significantly reduced 90-day survival following EL, with affected patients experiencing a 2.7-fold increased risk of mortality within the first 90 postoperative days compared to those without pulmonary complications (*p*_log-rank_ <0.0001, HR 2.714; 95% CI 2.006–3.672; Figure 1A). Preoperative factors independently associated with increased PPC risk also influenced 90-day mortality (Figures 1B,C). In both groups, lower ASA scores (≤3) were associated with improved survival (no-PPC group: *p*_log-rank_ = 0.0012; PPC group: *p*_log-rank_ = 0.0003; Figures 1B1,C1). A preoperative hemoglobin level above the 100 g/L threshold—previously established in risk models for non-cardiac surgical populations (8, 9, 33, 34)—was associated with a survival benefit; however, this effect was observed only in patients who developed PPCs (Figure 1C2).

As no established cutoff for CAR existed in this setting, we used the Youden index (29) to determine an optimal threshold of 1.07. Applying this cutoff revealed a significant survival difference in both the PPC and no-PPC groups (*p*_log-rank_ = 0.0153 and *p*_log-rank_ = 0.0013, respectively; Figures 1B3,C3).

Interestingly, while MDRO colonization had no impact on survival in the no-PPC group (Figure 1B4), it significantly worsened outcomes in patients with PPCs (*p*_log-rank_ = 0.0359, HR 1.458, 95% CI 0.990–2.146; Figure 1C4), with median 90-day survival being 20 days shorter in colonized patients compared to non-colonized patients (34 vs. 54 days, respectively).

### 3.5 MDRO colonization and antibiotic coverage in PPC patients

Each of the four identified independent risk factors for PPC development—ASA score, CAR, hemoglobin level, and MDRO colonization—also influenced survival in the PPC group. Following Miskovic and Lumb’s framework (9), these can be categorized into non-modifiable (ASA score, CAR) and modifiable factors (hemoglobin level, MDRO colonization). While colonization status cannot be changed perioperatively, empiric antibiotic therapy can be adjusted accordingly. Therefore, we conducted a detailed analysis of MDRO colonization in the PPC patient group.

**Figure 2:**
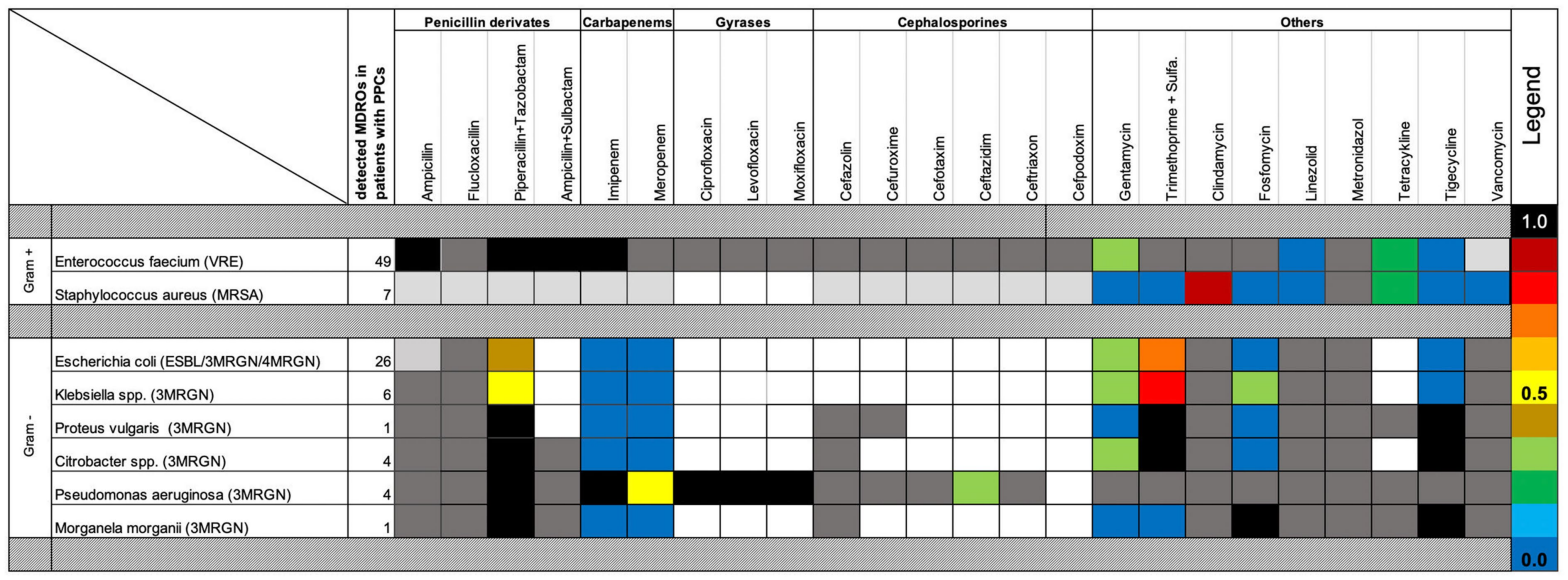
Antibiotic susceptibility and resistance of perioperatively detected MDROs in PPC patients. Resistance patterns to commonly used perioperative antibiotics are shown for multidrug-resistant organisms (MDROs) detected in patients with postoperative pulmonary complications (PPCs). Bacteria were categorized as Gram positive or Gram negative. Dark-gray boxes indicate intrinsic resistance; light-gray boxes indicate resistance implied by the MDRO definition. Blue shades denote the lowest resistance rates. Colored boxes represent the frequency of acquired antibiotic resistance.

VRE was the most frequently isolated MDRO, followed by MDR *Escherichia coli* (Table 4). Notably, 67.3% of MDROs were not adequately covered by empiric antibiotic regimens. Resistance profiles— including both intrinsic acquired antibiotic resistances—are detailed in Figure 2.

**Table 4:** Preoperative MDRO colonization in PPC patients: characteristics, empiric antibiotic coverage, and postoperative airway isolates.

Detected MDRO	Gram-positive MDROs		Gram-negative MDROs			Total
	Vancomycin-resistant *Enterococcus faecium* (VRE) (*n* = 49)	Methicillin-resistant *Staphylococcus aureus* (MRSA) (*n* = 7)	*Escherichia coli* species (ESBL/ 3 MRGN/ 4 MRGN) (*n* = 26)	*Klebsiella* species (3 MRGN) (*n* = 6)	Others (3 MRGN) (*n* = 10)	
Initial colonization						
Anal	44 (89.8%)	0 (0%)	17 (65.4%)	3 (50%)	5 (50%)	69 (70.4%)
Oral/nasal	1 (2%)	7 (100%)	9 (34.6%)	2 (33.3%)	4 (40%)	23 (23.5%)
Other	4 (8.2%)	0 (0%)	0 (0%)	1 (16.7%)	1 (10%)	6 (6.1%)
Covered by empiric antibiotic therapy						
Yes	10 (20.4%)	1 (14.3%)	11 (42.3%)	5 (83.3%)	5 (50%)	32 (32.7%)
No	39 (79.6%)	6 (85.7%)	15 (57.7%)	1 (16.7%)	5 (50%)	66 (67.3%)
Postoperative airway contamination						
No available sample	19 (38.8%)	3 (38.5%)	10 (38.5%)	3 (50%)	4 (40%)	39 (39.8%)
Sterile sample	8 (16.3%)	0 (0%)	1 (3.8%)	2 (33.3%)	0 (0%)	11 (11.2%)
Same MDRO identified	1 (2%)	0 (0%)	9 (34.6%)	1 (16.7%)	2 (20%)	13 (13.3%)
Other pathogen identified	21 (42.9%)	4 (57.1%)	6 (23.1%)	0 (0%)	4 (40%)	35 (35.7%)

Preoperative multidrug-resistant organisms (MDROs) in patients with postoperative pulmonary complications (PPCs), classified by Gram status and antibiotic-resistance profile. Coverage by the initially administered empiric antibiotic therapy and microbiological findings from postoperative airway sampling in the same patients were evaluated. VRE, vancomycin-resistant enterococci; MRSA, methicillin-resistant *Staphylococcus aureus*; ESBL, extended-spectrum β-lactamase-producing *Enterobacterales*; MRGN, multidrug-resistant Gram-negative bacteria.

Overall, the same MDRO identified in preoperative screening swabs was subsequently detected in 13.3% of postoperative tracheal or bronchial aspirates. Notably, Gram-negative MDROs exhibited a significantly higher rate of postoperative airway contamination compared to Gram-positive organisms. Specifically, 28.6% of Gram-negative MDROs detected preoperatively were later found in the upper airways of patients who developed PPCs following EL (Table 4).

## 4 Discussion

Our study highlights the substantial clinical burden of PPCs in patients undergoing EL, emphasizing their strong association with both short-term morbidity and 90-day mortality. Through the identification of modifiable and non-modifiable preoperative risk factors—specifically preoperative hemoglobin <10 g/dL and colonization with MDROs—our findings suggest opportunities for targeted interventions even within the narrow therapeutic window inherent to emergency settings.

The PPC rate in our cohort was 35.3%, aligning with the 20–40% range reported in earlier studies and slightly lower than the 48% observed in the ALPINE trial (2, 12). Prior work by Havens et al. (1) demonstrated that among all postoperative complications—including pulmonary, cardiac, septic, and wound-related—pulmonary complications were most strongly associated with the emergent nature of surgery, reinforcing the idea that both acute surgical pathology and the procedural trauma significantly heighten respiratory risk in this particularly vulnerable patient group.

To identify actionable factors linked to both acute surgical pathology and the procedural EL trauma, we employed PSM to control for non-modifiable demographic and preexisting health issues. Our analysis revealed ASA score, CAR, preoperative hemoglobin levels, and colonization with MDROs as independent predictors of PPC development. Importantly, all of these factors were also significantly associated with 90-day mortality among patients who developed PPCs, underscoring their clinical relevance beyond the immediate postoperative period.

The ASA classification remains the most widely used and accessible tool for perioperative risk stratification (35). Its simplicity makes it preferable to more complex scoring systems, and numerous studies—predominantly in elective surgery—have demonstrated its ability to predict postoperative morbidity, including the risk of PPCs (36, 37). However, its utility in emergency contexts is debated. As Horvath et al. (38) have pointed out, the ASA system lacks clear guidance on how acute physiological insults should be integrated into the preoperative status assignment. For instance, a patient with obesity and well-controlled diabetes would typically be assigned ASA II, but if presenting with sepsis, the patient may be reclassified as ASA IV-E. While such transitions are relatively straightforward, more nuanced clinical deterioration can introduce subjectivity into the scoring process, raising concerns about its consistency and predictive power in emergency settings.

Nonetheless, in our cohort, the ASA score remained one of the most powerful independent predictors of PPCs. Patients with ASA scores above 3 experienced significantly higher 90-day mortality, both in those who developed PPCs (*p* = 0.0003) and in those who did not (*p* = 0.0012)—suggesting that despite its limitations, the ASA score retains prognostic value even in acute care settings.

CAR, a composite biomarker that reflects systemic inflammation through the positive acute-phase protein CRP (39) and the negative acute-phase protein albumin, which is also associated with nutritional risk (40), has shown good performance as an outcome predictor in critical illness across many studies (30–32).

In a large retrospective study involving over 11,000 surgical patients, Oh et al. (31) demonstrated that CAR measured upon ICU admission independently predicted both 30-day and one-year mortality. In oncologic surgery, where hypoalbuminemia and systemic inflammation are common, elevated CAR has been predictive of increased postoperative complications, although pulmonary complications were not specifically addressed (41, 42). Notably, in non-surgical cohorts such as COVID-19 patients, higher CAR values on admission have been linked to reduced oxygen saturation and impaired pulmonary function parameters (SO₂, FEV₁%, and FVC%) (43).

In our study, the preoperative CAR emerged as a robust independent predictor of PPCs in EL. Using the Youden index (29), we identified an optimal CAR cut-off value of 1.07, which was predictive of 90-day survival regardless of whether a pulmonary complication occurred.

Mechanistically, the prognostic value of CAR is underpinned by the pathophysiological roles of its two components—CRP, a positive acute-phase reactant, and albumin, a negative acute-phase reactant. CRP, in its native pentameric form, dissociates upon encountering damaged cell membranes into monomeric subunits that exhibit potent pro-inflammatory and pro-coagulant properties (39). As shown by Fendl et al. (44), these monomeric CRP forms are trafficked via extracellular vesicles to distant organs, thereby amplifying systemic inflammation and contributing to remote organ injury, including lung damage.

Conversely, hypoalbuminemia reduces plasma oncotic pressure, facilitating fluid transudation into the alveolar space and promoting pulmonary edema. This condition has been independently associated with an elevated risk of acute respiratory distress syndrome (ARDS) in critically ill patients (45).

Although therapeutic strategies aimed at neutralizing CRP’s pro-inflammatory activity are under investigation, they have not yet advanced beyond experimental phases (46). Similarly, the efficacy of albumin supplementation to prevent ARDS remains inconclusive (47). Therefore, while CAR itself is not a directly modifiable parameter at present, it may serve as a valuable prognostic biomarker and a potential target for future therapeutic interventions in patients undergoing EL.

Preoperative anemia with a hemoglobin level below 100 g/L has been categorized as a modifiable risk factor for PPC development in the pivotal work of Miskovic and Lumb (9). Because intraoperative blood transfusion has also been observed to increase PPC risk, the authors recommend alternative treatment strategies—such as dietary measures or iron supplementation—depending on the underlying cause of the anemia (9). Such optimization, however, is naturally limited in emergency settings.

Currently, there is limited evidence regarding the significance and clinical consequences of preoperative colonization with MDROs in patients undergoing EL. When formulating clinical practice guidelines for perioperative antibiotic prophylaxis in patients colonized with Gram-negative MDROs, the European Society of Clinical Microbiology and Infectious Diseases (ESCMID) explicitly emphasized major gaps in the available evidence (48). As a result, the issued recommendations were primarily based on low-level data and restricted to select patient populations, such as those undergoing elective colorectal surgery (20, 48, 49), or solid organ transplantation (22, 48).

In elective colorectal surgery, preoperative colonization with MDROs has been associated with an increased risk of surgical site infections (20, 48, 49). Among solid organ transplant recipients, such colonization has been linked to more severe postoperative complications, including elevated rates of all-cause mortality, infection-related mortality, polymicrobial infections, and bloodstream infections (22, 48). In critically ill patients—regardless of surgical status—MDRO colonization at ICU admission has similarly been correlated with higher in-hospital mortality, even after adjusting for illness severity (50, 51).

These findings suggest that the clinical impact of MDRO colonization is particularly pronounced in immunocompromised or physiologically unstable patients. In such cases, delays in initiating appropriate antimicrobial therapy may substantially contribute to poor outcomes. In our study, PPC patients demonstrated a significantly higher prevalence of MDRO colonization prior to surgery (25%) compared to those without PPCs (13%). Importantly, this colonization was also associated with significantly decreased 90-day survival in the PPC group (*p* = 0.0359), whereas no such association was observed in patients without pulmonary complications—possibly reflecting the amplified inflammatory and infectious burden in the former.

Microbiological cultures from the upper airways were obtained in 60% of patients who developed PPCs. Interestingly, while patients with Gram-positive MDRO colonization typically developed respiratory infections with unrelated pathogens, 26.8% of those colonized with Gram-negative MDROs harbored the same organism in their postoperative airway samples. Despite this, only half of these patients had received empiric antibiotic coverage that adequately targeted these resistant pathogens. This raises an important question: could earlier, more tailored antimicrobial therapy reduce pulmonary complications and mortality in high-risk, colonized patients?

Although MDRO colonization emerged as an important preoperative predictor of PPCs in our analysis, routine microbiological screening and antimicrobial susceptibility testing are generally not available prior to emergency surgery—unless this information is already known from a previous hospitalization. Nevertheless, several rapid diagnostic platforms have shown the potential to identify MDRO colonization or infection within clinically actionable timeframes. These include MALDI-TOF/RAST (matrix-assisted laser desorption/ionization time-of-flight mass spectrometry with rapid antimicrobial susceptibility testing), multiplex culture-independent diagnostics (MCD), and point-of-care multiplex polymerase chain reaction (PCR) systems, which can provide results within approximately 0.5–7 h (52–54). Although these technologies are not yet widely available in routine emergency surgical practice, their broader future implementation may allow earlier detection of MDRO carriers and help guide more targeted perioperative antimicrobial strategies.

This study is subject to several limitations. Its retrospective design, while enabling real-world data collection, inherently restricts causal interpretation. Functional respiratory parameters and preoperative arterial blood gas analyses were not consistently available and therefore could not be included in the analysis. Microbiological airway sampling was performed only in patients with clinical indications rather than routinely, introducing a potential selection bias. As all data were collected at a tertiary university hospital, referral bias is also possible, which may limit the generalizability of our findings. Finally, regional variation in MDRO prevalence, resistance patterns, and antimicrobial stewardship practices may restrict the transferability of our results to other healthcare settings.

Despite these limitations, this study represents an important first step in systematically investigating the impact of PPCs and MDRO colonization in the high-risk EL population. By identifying both modifiable and non-modifiable risk factors associated with poor outcomes, our findings lay the groundwork for improved perioperative risk stratification and patient-centered management strategies. Given the increasing prevalence of antimicrobial resistance and the persistently high morbidity associated with PPCs in emergency surgery, further prospective, multicenter studies are urgently needed to validate these findings and inform evidence-based interventions tailored to this vulnerable patient cohort.

## 5 Conclusion

In summary, PPCs following EL are common and have a profound impact on patient outcomes, significantly increasing mortality. Our study identifies a high preoperative ASA score, an elevated preoperative CAR, low preoperative hemoglobin, and MDRO colonization as independent predictors of PPC development and as factors associated with increased 90-day mortality in patients with PPCs. Among these, preoperative colonization with Gram-negative MDROs may represent the most readily actionable risk factor, even in the constrained emergency setting. These findings underscore the importance of rigorous preoperative risk stratification and support future studies to evaluate whether patients at high risk of PPCs could benefit from antibiotic regimens tailored to their colonizing MDROs.

## Data Availability

The raw data supporting the conclusions of this article will be made available by the authors, without undue reservation.

## References

[1] Havens JM Peetz AB Do WS Cooper Z Kelly E Askari R . The excess morbidity and mortality of emergency general surgery. J Trauma Acute Care Surg. (2015) 78:306–11. doi: 10.1097/TA.0000000000000517 25757115

[2] Ylimartimo AT Nurkkala J Koskela M Lahtinen S Kaakinen T Vakkala M . Postoperative complications and outcome after emergency laparotomy: a retrospective study. World J Surg. (2023) 47:119–29. doi: 10.1007/s00268-022-06783-8, 36245004 PMC9726776

[3] Peden CJ Aggarwal G Aitken RJ Anderson ID Bang Foss N Cooper Z . Guidelines for perioperative care for emergency laparotomy: enhanced recovery after surgery (ERAS) society recommendations. Part 1-preoperative: diagnosis, rapid assessment and optimization. World J Surg. (2021) 45:1272–90. doi: 10.1007/s00268-021-05994-9, 33677649 PMC8026421

[4] Scott MJ Aggarwal G Aitken RJ Anderson ID Balfour A Foss NB . Consensus guidelines for perioperative care for emergency laparotomy: enhanced recovery after surgery (ERAS^®^) society recommendations. Part 2-intra- and postoperative care. World J Surg. (2023) 47:1850–80. doi: 10.1007/s00268-023-07020-6 37277507 PMC10241558

[5] Ceresoli M Braga M Zanini N Abu-Zidan FM Parini D Langer T . Enhanced perioperative care in emergency general surgery: the WSES position paper. World J Emerg Surg. (2023) 18:47. doi: 10.1186/s13017-023-00519-2, 37803362 PMC10559594

[6] Cusack B Buggy DJ. Anaesthesia, analgesia, and the surgical stress response. BJA Educ. (2020) 20:321–8. doi: 10.1016/j.bjae.2020.04.006, 33456967 PMC7807970

[7] Xiang B Jiao S Si Y Yao Y Yuan F Chen R. Risk factors for postoperative pneumonia: a case-control study. Front Public Health. (2022) 10:913897. doi: 10.3389/fpubh.2022.913897, 35875004 PMC9304902

[8] Canet J Gallart L Gomar C Paluzie G Vallès J Castillo J . Prediction of postoperative pulmonary complications in a population-based surgical cohort. Anesthesiology. (2010) 113:1338–50. doi: 10.1097/ALN.0b013e3181fc6e0a, 21045639

[9] Miskovic A Lumb AB. Postoperative pulmonary complications. Br J Anaesth. (2017) 118:317–34. doi: 10.1093/bja/aex002, 28186222

[10] Warner DO. Preventing postoperative pulmonary complications: the role of the anesthesiologist. Anesthesiology. (2000) 92:1467–72. doi: 10.1097/00000542-200005000-00037, 10781293

[11] Abbott TEF Fowler AJ Pelosi P Gama de Abreu M Møller AM Canet J . A systematic review and consensus definitions for standardised end-points in perioperative medicine: pulmonary complications. Br J Anaesth. (2018) 120:1066–79. doi: 10.1016/j.bja.2018.02.007, 29661384

[12] Watson X Chereshneva M Odor PM Ster IC Pan-London Perioperative Audit and Research Network (PLAN) Cecconi M. Adoption of lung protective ventilation in patients undergoing emergency laparotomy: the ALPINE study. A prospective multicentre observational study. Br J Anaesth. (2018) 121:909–17. doi: 10.1016/j.bja.2018.04.048, 30236253

[13] Fagevik Olsén M Hahn I Nordgren S Lönroth H Lundholm K. Randomized controlled trial of prophylactic chest physiotherapy in major abdominal surgery. Br J Surg. (1997) 84:1535–8. doi: 10.1111/j.1365-2168.1997.02828.x, 9393272

[14] Odor PM Bampoe S Gilhooly D Creagh-Brown B Moonesinghe SR. Perioperative interventions for prevention of postoperative pulmonary complications: systematic review and meta-analysis. BMJ. (2020) 368:m540. doi: 10.1136/bmj.m540, 32161042 PMC7190038

[15] Futier E Constantin JM Paugam-Burtz C Pascal J Eurin M Neuschwander A . A trial of intraoperative low-tidal-volume ventilation in abdominal surgery. N Engl J Med. (2013) 369:428–37. doi: 10.1056/NEJMoa1301082, 23902482

[16] Montravers P Veber B Auboyer C Dupont H Gauzit R Korinek AM . Diagnostic and therapeutic management of nosocomial pneumonia in surgical patients: results of the Eole study. Crit Care Med. (2002) 30:368–75. doi: 10.1097/00003246-200202000-00017, 11889312

[17] Fujita T Ishida Y Yanaga K. Impact of appropriateness of initial antibiotic therapy on outcome of postoperative pneumonia. Langenbecks Arch Surg. (2008) 393:487–91. doi: 10.1007/s00423-007-0271-5, 18176815

[18] Martin-Loeches I Rodriguez AH Torres A. New guidelines for hospital-acquired pneumonia/ventilator-associated pneumonia: USA vs. Europe. Curr Opin Crit Care. (2018) 24:347–52. doi: 10.1097/MCC.0000000000000535, 30063491

[19] Cillóniz C Dominedò C Torres A. An overview of guidelines for the management of hospital-acquired and ventilator-associated pneumonia caused by multidrug-resistant Gram-negative bacteria. Curr Opin Infect Dis. (2019) 32:656–62. doi: 10.1097/QCO.0000000000000596, 31567412

[20] Mehdorn M Kolbe-Busch S Lippmann N Moulla Y Scheuermann U Jansen-Winkeln B . Rectal colonization is predictive for surgical site infections with multidrug-resistant bacteria in abdominal surgery. Langenbecks Arch Surg. (2023) 408:230. doi: 10.1007/s00423-023-02961-x, 37301803 PMC10257639

[21] De Pastena M Paiella S Azzini AM Marchegiani G Malleo G Ciprani D . Preoperative surveillance rectal swab is associated with an increased risk of infectious complications in pancreaticoduodenectomy and directs antimicrobial prophylaxis: an antibiotic stewardship strategy? HPB. (2018) 20:555–62. doi: 10.1016/j.hpb.2017.12.002, 29336894

[22] Almohaya A Fersovich J Weyant RB Fernández García OA Campbell SM Doucette K . The impact of colonization by multidrug resistant bacteria on graft survival, risk of infection, and mortality in recipients of solid organ transplant: systematic review and meta-analysis. Clin Microbiol Infect. (2024) 30:1228–43. doi: 10.1016/j.cmi.2024.03.036, 38608872

[23] von Elm E Altman DG Egger M Pocock SJ Gøtzsche PC Vandenbroucke JP . Strengthening the reporting of observational studies in epidemiology (STROBE) statement: guidelines for reporting observational studies. BMJ. (2007) 335:806–8. doi: 10.1136/bmj.39335.541782.AD, 17947786 PMC2034723

[24] Kommission für Krankenhaushygiene und Infektionsprävention (KRINKO) beim Robert-Koch-Institut (RKI). Hygienemaßnahmen bei Infektionen oder Besiedelung mit multiresistenten gramnegativen Stäbchen. Bundesgesundheitsblatt Gesundheitsforschung Gesundheitsschutz. (2012) 55:1311–54. doi: 10.1007/s00103-012-1549-5 23011096

[25] World Health Organization. WHO Bacterial Priority Pathogens List, 2024: bacterial pathogens of public health importance to guide research, development and strategies to prevent and control antimicrobial resistance. Geneva: World Health Organization (2024).

[26] Elamin A Walker E Sugrue M Khalid SY Stephens I Lloyd A. Enhancing operative documentation of emergency laparotomy: a systematic review and development of a synoptic reporting template. World J Emerg Surg. (2023) 18:53. doi: 10.1186/s13017-023-00523-6, 38037125 PMC10688081

[27] Jammer I Wickboldt N Sander M Smith A Schultz MJ Pelosi P . Standards for definitions and use of outcome measures for clinical effectiveness research in perioperative medicine: European perioperative clinical outcome (EPCO) definitions: a statement from the ESA-ESICM joint taskforce on perioperative outcome measures. Eur J Anaesthesiol. (2015) 32:88–105. doi: 10.1097/EJA.0000000000000118, 25058504

[28] Dindo D Demartines N Clavien PA. Classification of surgical complications: a new proposal with evaluation in a cohort of 6336 patients and results of a survey. Ann Surg. (2004) 240:205–13. doi: 10.1097/01.sla.0000133083.54934.ae, 15273542 PMC1360123

[29] Ruopp MD Perkins NJ Whitcomb BW Schisterman EF. Youden index and optimal cut-point estimated from observations affected by a lower limit of detection. Biom J. (2008) 50:419–30. doi: 10.1002/bimj.200710415, 18435502 PMC2515362

[30] Ranzani OT Zampieri FG Forte DN Azevedo LC Park M. C-reactive protein/albumin ratio predicts 90-day mortality of septic patients. PLoS One. (2013) 8:e59321. doi: 10.1371/journal.pone.0059321, 23555017 PMC3595283

[31] Oh TK Song IA Lee JH. Clinical usefulness of C-reactive protein to albumin ratio in predicting 30-day mortality in critically ill patients: a retrospective analysis. Sci Rep. (2018) 8:14977. doi: 10.1038/s41598-018-33361-7, 30297724 PMC6175848

[32] Park JE Chung KS Song JH Kim SY Kim EY Jung JY . The C-reactive protein/albumin ratio as a predictor of mortality in critically ill patients. J Clin Med. (2018) 7:333. doi: 10.3390/jcm7100333, 30297655 PMC6210319

[33] Baron DM Hochrieser H Posch M Metnitz B Rhodes A Moreno RP . Preoperative anaemia is associated with poor clinical outcome in non-cardiac surgery patients. Br J Anaesth. (2014) 113:416–23. doi: 10.1093/bja/aeu098, 24829444

[34] Lee JY Lee SH Jung MJ Lee JG. Perioperative risk factors for in-hospital mortality after emergency gastrointestinal surgery. Medicine. (2016) 95:e4530. doi: 10.1097/MD.0000000000004530, 27583863 PMC5008547

[35] Sankar A Johnson SR Beattie WS Tait G Wijeysundera DN. Reliability of the American Society of Anesthesiologists physical status scale in clinical practice. Br J Anaesth. (2014) 113:424–32. doi: 10.1093/bja/aeu100, 24727705 PMC4136425

[36] Neto AS da Costa LGV Hemmes SNT Canet J Hedenstierna G Jaber S . The LAS VEGAS risk score for prediction of postoperative pulmonary complications: an observational study. Eur J Anaesthesiol. (2018) 35:691–701. doi: 10.1097/EJA.0000000000000845, 29916860 PMC7450515

[37] Hackett NJ De Oliveira GS Jain UK Kim JY. ASA class is a reliable independent predictor of medical complications and mortality following surgery. Int J Surg. (2015) 18:184–90. doi: 10.1016/j.ijsu.2015.04.079, 25937154

[38] Horvath B Kloesel B Todd MM Cole DJ Prielipp RC. The evolution, current value, and future of the American Society of Anesthesiologists physical status classification system. Anesthesiology. (2021) 135:904–19. doi: 10.1097/ALN.0000000000003947, 34491303

[39] Sproston NR Ashworth JJ. Role of C-reactive protein at sites of inflammation and infection. Front Immunol. (2018) 9:754. doi: 10.3389/fimmu.2018.00754, 29706967 PMC5908901

[40] Evans DC Corkins MR Malone A Miller S Mogensen KM Guenter P . The use of visceral proteins as nutrition markers: an ASPEN position paper. Nutr Clin Pract. (2021) 36:22–8. doi: 10.1002/ncp.10588, 33125793

[41] Ge X Cao Y Wang H Ding C Tian H Zhang X . Diagnostic accuracy of the postoperative ratio of C-reactive protein to albumin for complications after colorectal surgery. World J Surg Oncol. (2017) 15:15. doi: 10.1186/s12957-016-1092-1, 28069031 PMC5223565

[42] Lee JW Sharma AR Lee SS Chun WJ Kim HS. The C-reactive protein to albumin ratio predicts postoperative complication in patients who undergo gastrectomy for gastric cancer. Heliyon. (2020) 6:e04220. doi: 10.1016/j.heliyon.2020.e04220, 32577578 PMC7303550

[43] Afsin DE Kerget B. Evaluation of the relationship between CRP/albumin ratio and pulmonary function parameters in patients with post-acute COVID-19. Clin Lab. (2022) 68:211102. doi: 10.7754/Clin.Lab.2021.211102, 35975504

[44] Fendl B Weiss R Eichhorn T Linsberger I Afonyushkin T Puhm F . Extracellular vesicles are associated with C-reactive protein in sepsis. Sci Rep. (2021) 11:6996. doi: 10.1038/s41598-021-86489-4, 33772103 PMC7997920

[45] McNeil JB Jackson KE Wang C Siew ED Vincz AJ Shaver CM . Linear association between hypoalbuminemia and increased risk of acute respiratory distress syndrome in critically ill adults. Crit Care Explor. (2021) 3:e0527. doi: 10.1097/CCE.0000000000000527, 34549190 PMC8443821

[46] Rizo-Téllez SA Sekheri M Filep JG. C-reactive protein: a target for therapy to reduce inflammation. Front Immunol. (2023) 14:1237729. doi: 10.3389/fimmu.2023.1237729, 37564640 PMC10410079

[47] Uhlig C Silva PL Deckert S Schmitt J de Abreu MG. Albumin versus crystalloid solutions in patients with the acute respiratory distress syndrome: a systematic review and meta-analysis. Crit Care. (2014) 18:R10. doi: 10.1186/cc13187, 24405693 PMC4056106

[48] Righi E Mutters NT Guirao X Del Toro MD Eckmann C Friedrich AW . ESCMID/EUCIC clinical practice guidelines on perioperative antibiotic prophylaxis in patients colonized by multidrug-resistant Gram-negative bacteria before surgery. Clin Microbiol Infect. (2023) 29:463–79. doi: 10.1016/j.cmi.2022.12.012, 36566836

[49] Nutman A Temkin E Harbarth S Carevic B Ris F Fankhauser-Rodriguez C . Personalized ertapenem prophylaxis for carriers of extended-spectrum β-lactamase-producing Enterobacteriaceae undergoing colorectal surgery. Clin Infect Dis. (2020) 70:1891–7. doi: 10.1093/cid/ciz524, 31613316

[50] McConville TH Sullivan SB Gomez-Simmonds A Whittier S Uhlemann AC. Carbapenem-resistant enterobacteriaceae colonization (CRE) and subsequent risk of infection and 90-day mortality in critically ill patients, an observational study. PLoS One. (2017) 12:e0186195. doi: 10.1371/journal.pone.0186195, 29023567 PMC5638409

[51] Dautzenberg MJ Wekesa AN Gniadkowski M Antoniadou A Giamarellou H Petrikkos GL . The association between colonization with carbapenemase-producing enterobacteriaceae and overall ICU mortality: an observational cohort study. Crit Care Med. (2015) 43:1170–7. doi: 10.1097/CCM.0000000000001028, 25882764 PMC4431676

[52] Pariona JGM Oliveira FA Scoton PH Barrón-Pastor HJ Pariona EMM Zaccariotto TR . Rapid diagnostic of multidrug-resistant sepsis pathogens directly from blood culture bottles using MALDI-TOF and the EUCAST RAST. Diagn Microbiol Infect Dis. (2024) 109:116247. doi: 10.1016/j.diagmicrobio.2024.116247, 38484476

[53] Kuchibiro T Hirano A Ogasawara S Nakamura T. The microcolony detection method (MCD), a simple and rapid screening test for antimicrobial resistance bacteria on positive blood cultures. Heliyon. (2020) 6:e05494. doi: 10.1016/j.heliyon.2020.e05494, 33241155 PMC7672289

[54] Marxreiter S Marino J Callan K Hargrave J Alston T Fauntleroy K . Rapid detection of Gram-negative antimicrobial resistance determinants directly from positive blood culture broths using a multiplex PCR system. J Clin Microbiol. (2025) 63:e0038425. doi: 10.1128/jcm.00384-25, 41117625 PMC12607888

